# The SH2 domain and kinase activity of JAK2 target JAK2 to centrosome and regulate cell growth and centrosome amplification

**DOI:** 10.1371/journal.pone.0261098

**Published:** 2022-01-28

**Authors:** Aashirwad Shahi, Jacob Kahle, Chandler Hopkins, Maria Diakonova

**Affiliations:** Department of Biological Sciences, University of Toledo, Toledo, OH, United States of America; Hungarian Academy of Sciences, HUNGARY

## Abstract

JAK2 is cytokine-activated non-receptor tyrosine kinase. Although JAK2 is mainly localized at the plasma membrane, it is also present on the centrosome. In this study, we demonstrated that JAK2 localization to the centrosome depends on the SH2 domain and intact kinase activity. We created JAK2 mutants deficient in centrosomal localization ΔSH2, K882E and (ΔSH2, K882E). We showed that JAK2 WT clone strongly enhances cell proliferation as compared to control cells while JAK2 clones ΔSH2, K882E and (ΔSH2, K882E) proliferate slower than JAK2 WT cells. These mutant clones also progress much slower through the cell cycle as compared to JAK2 WT clone and the enhanced proliferation of JAK2 WT cells is accompanied by increased S −> G2 progression. Both the SH2 domain and the kinase activity of JAK2 play a role in prolactin-dependent activation of JAK2 substrate STAT5. We showed that JAK2 is an important regulator of centrosome function as the SH2 domain of JAK2 regulates centrosome amplification. The cells overexpressing ΔSH2 and (ΔSH2, K-E) JAK2 have almost three-fold the amplified centrosomes of WT cells. In contrast, the kinase activity of JAK2 is dispensable for centrosome amplification. Our observations provide novel insight into the role of SH2 domain and kinase activity of JAK2 in centrosome localization of JAK2 and in the regulation of cell growth and centrosome biogenesis.

## Introduction

JAK2 is a ubiquitously expressed non-receptor protein tyrosine kinases. JAK2 is activated by a variety of cytokines (including growth hormone, interferon gamma (IFNγ and prolactin (PRL), growth factors (including EGF and PDGF), and GPCR ligands and mediates signaling cascades that regulate cell survival, proliferation, and development (reviewed in [1, 2]). JAK2 plays a role in aging, inflammation, hematopoiesis, bone disorders, neurodegenerative diseases like Alzheimer’s disease, coagulation, thrombosis, ischemia and other cardiovascular events and malignant transformation of blood cells. Similarly, hyperactive JAK2-signaling is a hallmark of immune diseases (reviewed in [3]). JAK2 activates the Signal Transducers and Activators of Transcription (STAT) proteins, which then translocate to the nucleus to activate transcription of various target genes. Three mechanisms of JAK2 activation at the genetic level have been implicated in hematologic malignancies: point mutations, translocations and amplifications (reviewed in [4])). Thus, a V617F gain-of-function point mutation within the pseudokinase domain is the cause of ~90% of all polycythemia vera cases and ~50% of all essential thrombocythemia and primitive myelofibrosis cases [5, 6]. JAK2/STAT signaling is also activated in solid tumors (breast, prostate, melanomas and others) [7]. Thus, JAK2 can be considered as an important target for small molecule inhibitors [8].

Although JAK2 is involved in a wide range of human pathologies, the comprehensive mechanism of JAK2 regulation is not well defined. For example, it is unclear how a single point mutation can lead to several different disorders. Even fundamental question about subcellular localization of JAK2 is controversial. JAK2 localizes to the plasma membrane [9, 10], cytoplasm [10–13], and the endoplasmic reticulum [14]. Many (reviewed in [15]) but not all [16] have confirmed nuclear localization of JAK2. Moreover, JAK2 directly phosphorylates α/β tubulin and associates with microtubular (MT) cytoskeleton [17]. We have previously shown that activated JAK2 is a component of the mother centriole, where it partially co-localizes with the centriolar protein ninein and this localization depends on phase of the cell cycle [18]. We have also demonstrated that JAK2 plays a role in such ninein-dependent function as the proper attachment of MT to the mother centriole and that the absence of JAK2 causes multiple mitotic defects [18].

Centrosomes function as the main MT-organizing center (MTOC) regulating MT-based processes, notably intracellular transport and organelle distribution, cell shape and migration, bipolar spindle formation and cell division. Deregulation of centrosome structure and number is implicated in chromosomal instability and carcinogenesis [19–21]. Mutations in genes coding for centrosomal proteins have been causally linked to human disease, notably ciliopathies and brain diseases. However, the role of centrosomes extends well beyond that of important MT organizers. Centrosomes also function as coordination centers in eukaryotic cells, at which specific cytoplasmic proteins interact at high concentrations and important cell decisions are made. Nearly 600 proteins are concentrated at centrosomes, include cyclins, various kinases, phosphatases, signaling substrates and cell cycle regulators [22–24]. It has been proposed that centrosomes may function as a hub or solid base for the integration of signaling pathways [22, 25–30]. Some cytoplasmic tyrosine kinases directly target the centrosome in the course of their normal functions [31–33]. Our previously found localization of JAK2 to the centrosome also supports the idea that the centrosome might anchor different members of signal transduction pathways and serves as a centralized control center to regulate different cell functions [18].

In this study, we demonstrate that JAK2 localization to the centrosome depends on its SH2 domain and an intact kinase activity. The mutant of JAK2, lacking both the SH2 domain and the kinase activity (ΔSH2, K882E mutant) fails to localize to the centrosome. The same domains of JAK2 regulate cell growth. Thus, overexpression of JAK2 WT strongly promotes PRL-dependent cell proliferation while the effect of the overexpression of mutants, deficient in centrosomal localization (ΔSH2; K882E, and (ΔSH2, K882E) mutants) on cell proliferation is much weaker. These data are supported by the analysis of cell cycle. We show that the enhanced proliferation of JAK2 WT clone is accompanied by increased S −> G2 progression of these cells and that this effect depends on the SH2 domain and kinase activity of JAK2. JAK2 mutant clones (ΔSH2, K-E and (ΔSH2, K-E)) progress much slower through the cell cycle as compared to JAK2 WT clone, demonstrating S-phase delay. We also show that JAK2 is an important regulator of centrosome function because the SH2 domain of JAK2 participates in the regulation of centrosome amplification. The cells overexpressing JAK2 ΔSH2 and (ΔSH2, K-E) mutants have almost three-fold the amplified centrosomes of WT cells. Interestingly, kinase activity of JAK2 is not necessary for this. Our data suggest that the SH2 domain and the kinase activity of JAK2 play a role in the centrosomal localization of JAK2 and in regulation of cell growth and centrosome amplification.

## Materials and methods

### Cell culture

COS-7 (ATCC #CRL-1651), MCF-7 (ATCC # HTB-22) and 293T/17 (ATCC# CRL-11268) cells were purchased from the American Type Culture Collection. The cells were cultured in DMEM with sodium pyruvate supplemented with 10% FBS (Thermo Fisher Scientific, Gibco) and antibiotics. To generate stable COS-7 and MCF-7 clones overexpressing EGFP, EGFP-tagged JAK2 WT, or JAK2 mutants, we used 293T cells for virus production. The cells were transfected with pLV[Exp]-Neo-CMV>EGFP9 vector with packaging vectors psPAX2 (#12260, Addgene) and pMD2.G (#12259, Addgene) in a 4:3:1 ratio using a modification of the polyethylenimine method [34]. The medium was replaced with fresh medium in 12 hrs. In 48 and 72 hrs the virus broth was collected, filtered with a 45 μm filter, aliquoted and stored at -80°C. The COS-7 and MCF-7 cells were infected with lentiviruses supplemented with polybrene (final concentration of 8 μg/ml) and cultured at 37°C. The next day, the medium was replaced with fresh complete medium supplemented with 500 μg/ml G418 (Goldbio Inc.). Clonal cell lines were isolated by dilution and expanded, and at least six clonal lines were examined for exogenous expression by anti-GFP immunoblot.

### Plasmids and antibodies

cDNAs encoding EGFP-tagged JAK2 WT (mJAK2, NM-008413.2) and all JAK2 mutants were created by VectorBuilder Inc. in pLV[Exp]-Neo-CMV>EGFP9(ns) lentivirus gene expression vector (3^rd^ generation). Primary antibodies (AB) used in this study were rabbit monoclonal anti-JAK2 (D2E12) XP (Cell Signaling Technology Cat# 3230, RRID:AB_2128522; 1:100 dilution) and anti-pSTAT5 (Tyr694) (C11C5) (Cell Signaling Technology Cat# 9359, RRID:AB_823649; 1:500 dilution), mouse monoclonal anti-centrin 1(20H5) (Millipore Cat# 04–1624, RRID:AB_10563501; 1:1000 dilution), mouse monoclonal anti-γ-tubulin (GTU-88) (Sigma-Aldrich Cat# T6557, RRID:AB_477584; 1:1000 dilution), mouse monoclonal anti-GFP (B-2) (Santa Cruz Biotechnology Cat# sc-9996, RRID:AB_627695; 1:100 dilution), rabbit polyclonal anti-pHistone H3 (Ser10) (Cell Signaling Technology Cat# 9701, RRID:AB_331535; 1:200 dilution). Secondary AB were goat-anti-mouse and goat-anti-rabbit conjugated with either Alexa Fluor-594 or Alexa Fluor 488 (Thermo Fisher Scientific; 1:200 dilution for both).

**Immunoblotting** was performed as described in ref. [35].

**Immunocytochemistry** was performed as we described previously [18]. Briefly, for JAK2 localization, the COS-7 cells were plated on coverslips, infected with lentivirus in the presence of polybrene and fixed in 72 hrs with 4% formalin for 10 min followed by ice-cold methanol for 5 min. For detection of endogenous JAK2, the cells were rapidly permeabilized with 0.5% Triton X-100 in MT stabilizing buffer (MTSB; 0.1M PIPES [pH 6.9], 1mM EGTA, and 4M glycerol) for 1 min, washed with MTSB for 2 min, fixed in 20°C methanol for 7 min, and rehydrated in TBS (10mMTris [pH 7.5], 150mMNaCl) containing 0.1% bovine serum albumin for 10 min. The cells were incubated with blocking buffer (2% goat serum), incubated with anti-JAK2 AB. For centrosome localization, the cells were incubated with anti-centrin 1(20H5). Staining by secondary antibody reagent alone was negligible. DAPI (4`,6`-diamidino-2-phenylindole) (Invitrogen) was used for DNA staining. Mitotic cells were defined by cell morphology, centrosomal separation, and the state of the chromosomes (DNA staining), which were supported by the mitotic marker (anti-pHistone H3 (Ser10)) staining. Cell imaging was performed an Olympus microscope IX81 using a 60X/1.4 numerical aperture (NA) and X100/1.4 UPLSAPO objective lens. Confocal imaging was performed with an inverted Leica TCS SP8 laser scanning confocal microscope. All confocal images are maximal-intensity projections. All experiments were repeated at least 3 times with n≥ 50 cells quantified every time for each condition.

### Proliferation assay and cell cycle analysis

COS-7 (2500 cells/24-well plate) and MCF-7 (2 000 cells/24-well plate) were plated, washed in phosphate buffered saline (PBS) and deprived in DMEM supplemented with 1% FBS for 48 hrs. The cells were treated with 500 ng/ml PRL (MCF-7 cells), 30 ng/ml EGF (COS-7 cells) or vehicle, harvested in 4 days and counted using a hemocytometer. For cell cycle analysis, MCF-7 clones were plated at 15X10^4^ density in 6-well plate, synchronized on G1/S boundary by treatment with 2mM thymidine for 24 hrs, washed with PBS and incubated with DMEM supplemented with 1%FBS and 500 ng/ml PRL for indicated time points. Cells were trypsinized, pelleted, fixed with 70% ethanol and stored at -20°C overnight. The cells were then pelleted, washed with PBS, and incubated in propidium iodine (50 μg/ml) and RNASE A (100 μ/ml) for 30 min. The DNA content was analyzed with Becton Dickson FACScalibur fluorescence activated cell sorter. The percentage of cells in each phase of the cell cycle were calculated by FlowJo software. All experiments were repeated at least 3 times.

### STAT5 activation

MCF-7 clones were plated on the coverslips, deprived of serum for 2 days in deprivation medium (2% BSA in DMEM), treated with 500 ng/ml PRL for 0, 5, 10, 20, 40 and 60 min and fixed with ice-cold methanol for 10 min. After blocking with 2% goat serum, the cells were immunolabeled with anti-pSTAT5 AB following by goat-anti-rabbit-Alexa Fluor-594 AB and DAPI. Images of 50 cells per each condition were taken with the same light intensity to calculate the nuclear-to-cytosol fluorescence intensity ratios. The mean intensities of neighboring cytosolic and nuclear regions (approximately 5 mm^2^ each) were calculated using ImageJ and expressed as nuclear-to-cytosol ratios (N/C; [36]). The N/C ratio is 1 for equal fluorescence in the nucleus and cytosol, N/C >1 for prominent nuclear accumulation, and N/C < 1 for mostly cytoplasmic fluorescence. All experiments were repeated 3 times with 50 cells assessed every time for each condition.

**Statistical analysis** was performed as we described previously [18]. Data from at least three independent experiments per condition were pooled and analyzed using one-way analysis of variance (ANOVA) plus Tukey’s honestly significant difference (HSD) test. When individual experiments were analyzed, the results were indistinguishable from those obtained from the pooled data. Differences were considered statistically significant at a *P* value of < 0.05. Results are expressed as means ± standard errors (SE).

## Results

### JAK2 localization to the centrosome depends on SH2 domain and an intact kinase activity

**Fig 1:**
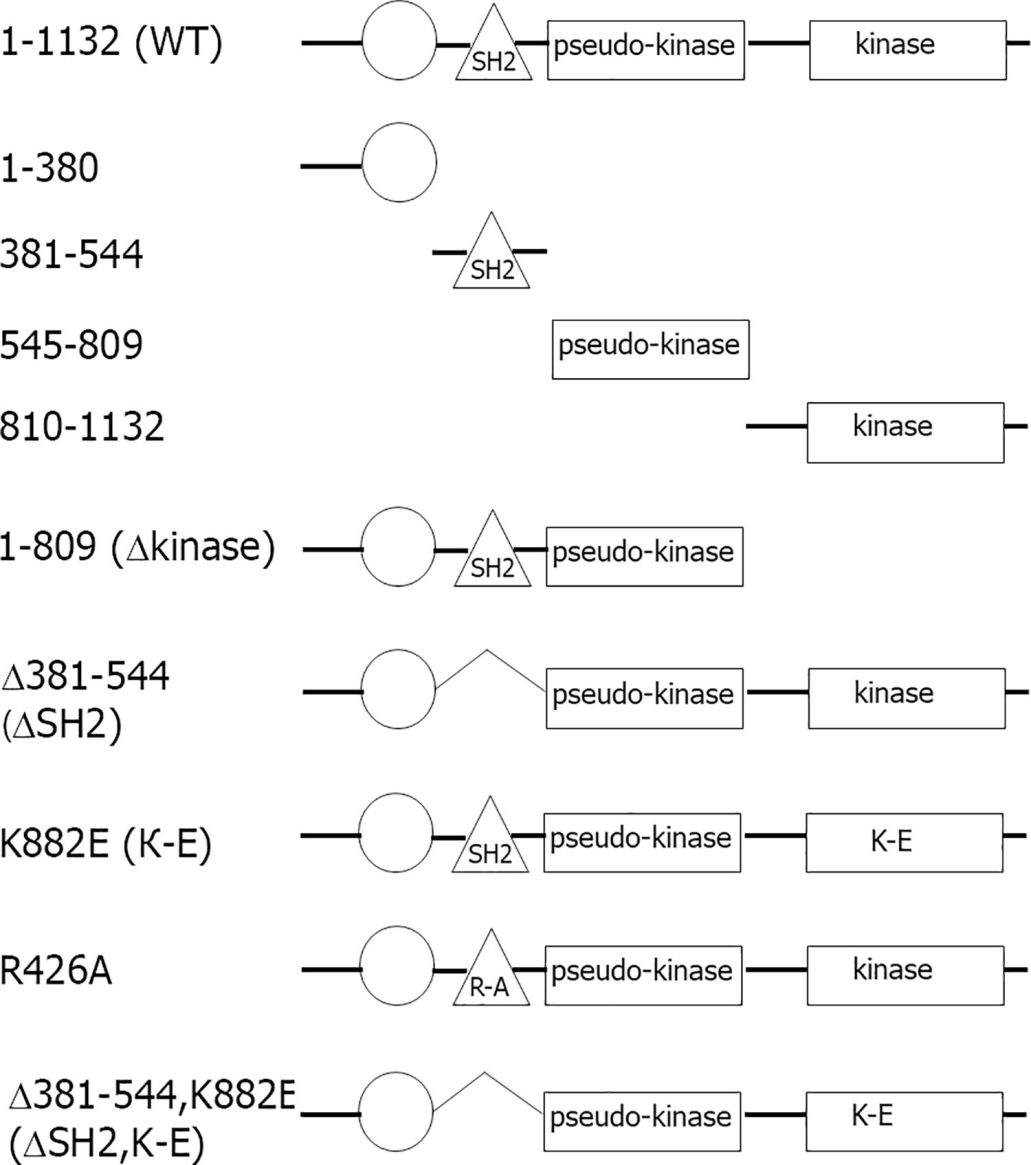
Schematic representation of wild type and mutant forms of mouse JAK2 used in this study. *Circle* represents FERM domain (AA 37–380). The *triangle* represents an SH2 domain (AA 401–482) and the *rectangles* represents pseudo-kinase (AA 545–809) and kinase (AA 849–1124) domains.

**Fig 2:**
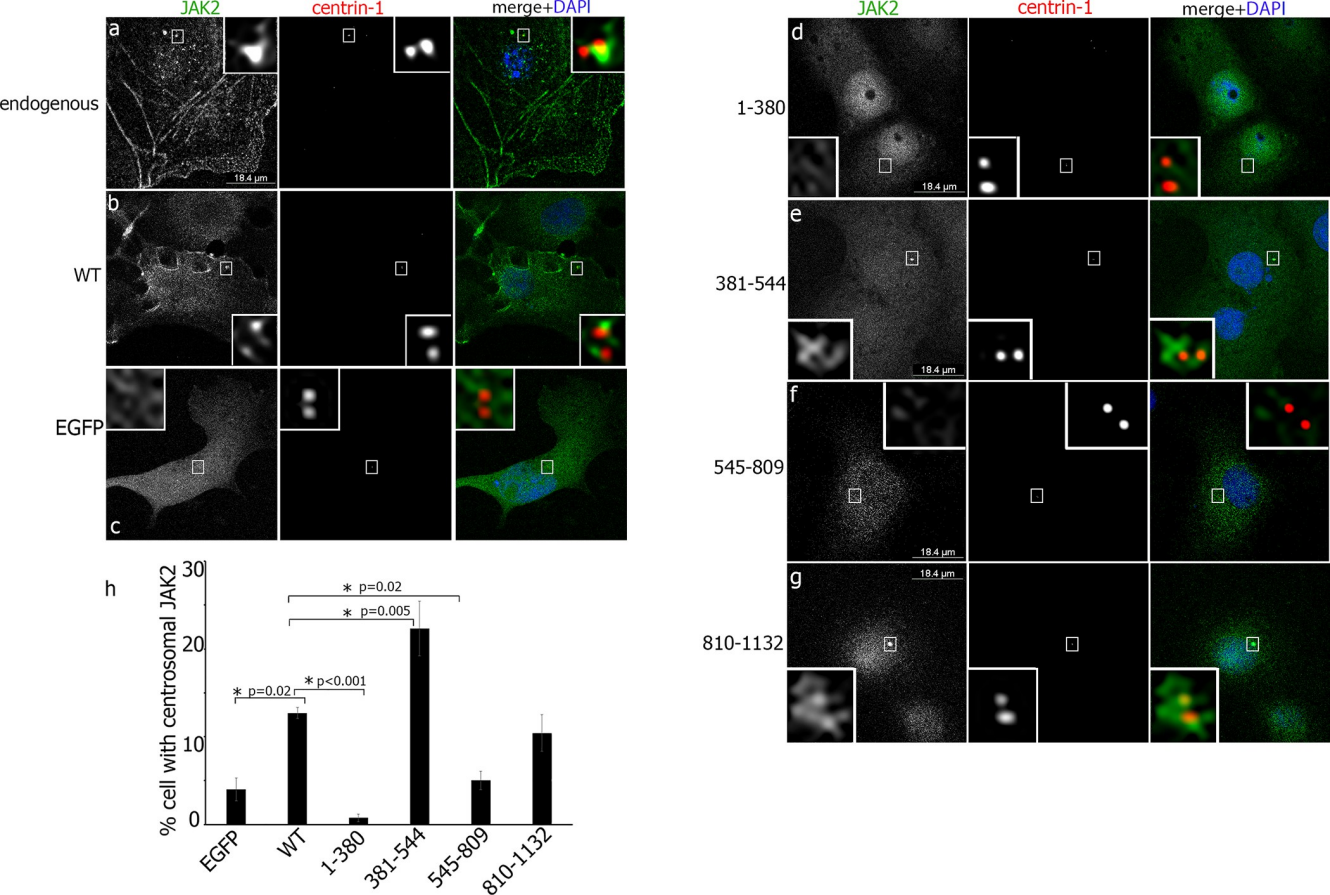
Centrosomal localization of JAK2 depends on both the SH2 domain and the intact kinase domain. COS-7 cells transiently overexpressing EGFP (**c**), WT (**b**) or the indicated forms of EGFP-JAK2 (**d-g**; green or yellow in the merged image) were immunolabeled with anti-centrin-1 AB (red in the merged image). DAPI stains cell nuclei (blue in the merge image). Anti-JAK2 AB were used to localize the endogenous JAK2 in non-transfected COS-7 cells (**a**) The insets show enlarged centrosomes. (**h**) The number of EGFP-positive cells with centrosomal JAK2 were scored and plotted. 50 cells were assessed in each experiment for each type of transfection. N = 9 for EGFP-expressed cells, n = 6 for WT and (381–544) mutant, n = 8 for (1–380) and (545–809) mutants and n = 7 for (810–1132) mutant. Bars represent mean ±SE. *, P< 0.05 compared with WT cells.

In order to identify which region of JAK2 targets it to the centrosome, we created a panel of truncated mutants. We generated 4 truncations of JAK2, encoding the N-terminal and FERM domain (amino acids (AA) 1–380), the central part with the SH2 domain (AA 381–544), the pseudokinase domain (AA 545–809) and the C-terminus with kinase domain (AA 810–1132) (**Fig 1**). All fragments, as well as wild-type JAK2, were fused with EGFP, confirmed by sequencing and their sizes and expression were confirmed by Western blotting. We transiently transfected the WT and the truncated mutants of EGFP-tagged JAK2 in COS-7 cells. The cells were also immunolabelled for centrin-1 to identify both centrioles. As expected, endogenous JAK2, detected by anti-JAK2 (green in **Fig 2A**), localized to one centriole labeled with anti-centrin-1 (red in **Fig 2A**, merge image). When WT JAK2 fused with EGFP was transiently expressed in COS-7 cells, it localized to the centrosome (**Fig 2B**) in contrast to the localization of EGFP protein, which was diffuse throughout the cytoplasm (**Fig 2C**). It is noteworthy that, unlike endogenous protein, exogenously expressed JAK2 demonstrated a tendency to localize to both centrioles. The same phenomenon was described earlier for the centrosomal protein CEP19 [37]. When we overexpressed truncated mutants of JAK2, both the SH2-domain-containing mutant (381–544) (**Fig 2E**) and the kinase-domain-containing mutant (810–1132) (**Fig 2G**) demonstrated centrosomal localization of these constructs, while FERM-domain-containing (1–380) (**Fig 2D**) and pseudo-kinase domain (545–809) (**Fig 2F**) distributed diffusively in the cytoplasm. Quantification of the percentage of cells with centrosomal localization of JAK2 mutants confirmed that WT, (381–544) and (810–1132) mutants localized to centrosomes while EGFP, (1–380) and (545–809) mutants failed to do it (**Fig 2H**). Interestingly, the mutant (381–544), containing the SH2 domain, demonstrated almost twice more cells with centrosomal localization as compared to the cells with overexpressed WT JAK2, suggesting that full length JAK2 may have a site that inhibits the interaction of WT JAK2 with the centrosome, for example, by masking critical amino acids in the SH2 domain. The data presented in Fig 2 suggest that JAK2 has two regions that play a role in the centrosomal localization: AA 381–544 and AA 810–1132. The first region has the SH2 domain and the second one has the kinase domain.

**Fig 3:**
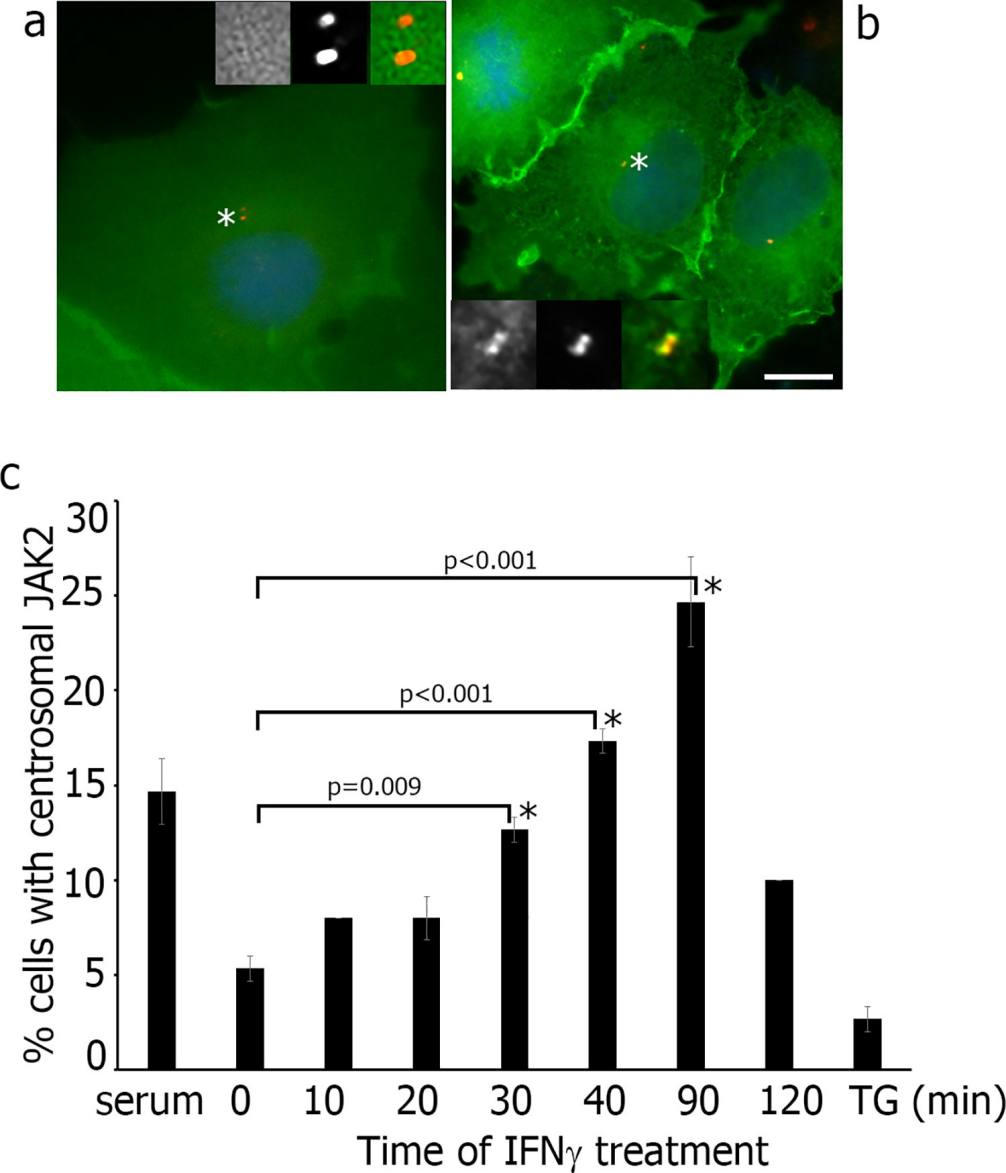
IFNγ treatment causes re-localization of JAK2 to centrosome. (**a, b**) COS-7 cells overexpressing EGFP-WT JAK2 (green) were serum deprived, treated with IFNγ, fixed and labeled with anti-centrin 1 (red) for centrosome localization. Image in (a) represents centrosome which is not labeled for JAK2 and image in (b) represents the centrosome labeled with JAK2. (**c**) The number of EGFP-positive cells with centrosomal JAK2 (as in (b)) were scored and plotted. Cells grown in serum were used as a positive control. JAK2 was inhibited by incubation with 50 mM of TG 101209 for 48 hrs followed by treatment with IFNγ for 90 min (last right bar). 50 cells were assessed in each experiment for each time point. N = 3. Bars represent mean ±SE. *, P< 0.05 compared with untreated cells (0 min). Scale bar 8 μm.

We sought to determine whether the intact amino acid sequence of kinase domain or kinase activity by itself is important for centrosomal localization of JAK2. To test whether the kinase activity of JAK2 participates in the centrosomal localization, we treated WT JAK2 overexpressing COS-7 cells with IFNγ, which activates JAK2 (reviewed in [38]) (**Fig 3A and 3B**). The treatment of COS-7 cells with IFNγ has been described previously (for example, [39, 40]). Quantification of serum-deprived COS-7 cells, treated with IFNγ for different time points, revealed that it takes 90 minutes to get the maximal amount of cells with centrosomal localization of JAK2 (**Fig 3C**). Next, we inhibited JAK2 by a potent JAK2 inhibitor TG 101209. This small molecule selectively inhibits JAK2 an *in vitro* and *in vivo* with significantly less activity against other tyrosine kinases [41]. We showed that incubation of the cells with JAK2 inhibitor TG 101209 (0.5 mM) for 48 hours led to dramatical decrease the amount of cells with centrosomal JAK2 after 90 min of IFNγ treatment (**Fig 3C**). These data suggest that kinase activity of JAK2 plays a role in its centrosomal localization.

**Fig 4:**
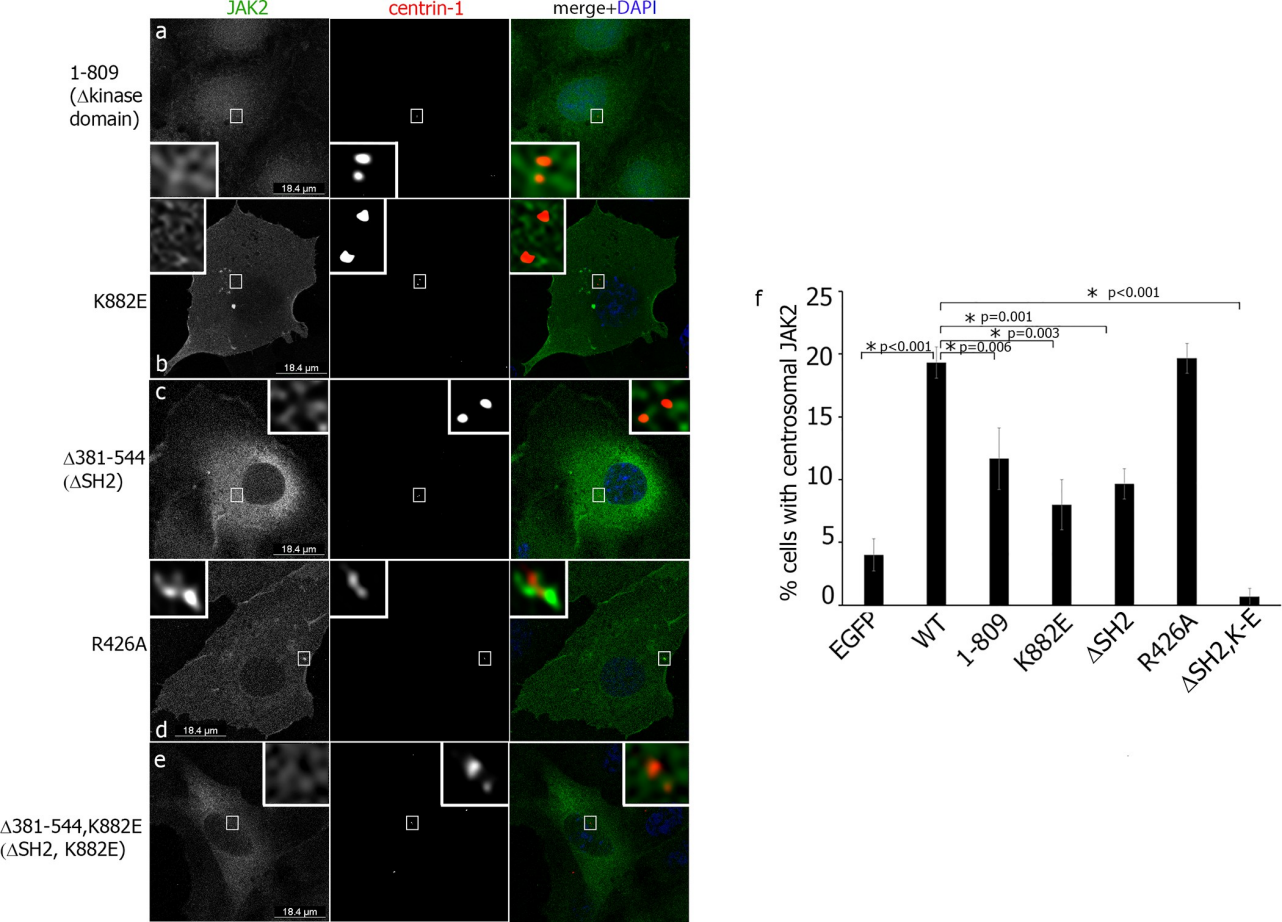
Both SH2 domain-containing amino acids 381–544 and kinase activity are required for centrosomal localization of JAK2. (**a-e**) COS-7 cells transiently overexpressing indicated forms of EGFP-JAK2 mutants (green in the merged image) were immunolabeled with anti-centrin-1 AB (red in the merged image). DAPI stains cell nuclei (blue in the merge image) The insets show enlarged centrosomes. (**f**) The number of EGFP-positive cells with centrosomal JAK2 were scored and plotted. 50 cells were assessed in each experiment for each type of transfection. N = 9 for EGFP and WT cells, n = 6 for (1–809), (Δ381–544) and R425 mutants. N = 3 for (1–809), K882E and (Δ381–544; K822E) mutants. Bars represent mean ±SE. *, P< 0.05 compared with WT cells.

Next, we created a panel of mutants deficient in centrosomal targeting of JAK2. All mutant constructs were fused with EGFP, confirmed by sequencing and their localization was assessed by immunofluorescence analysis (**Fig 4**). When we deleted amino acids 381–544 (Δ381–544 mutant lacking the SH2 domain; ΔSH2) (**Fig 4C**) or amino acids 810–1132 (1–809 mutant lacking the kinase domain) (**Fig 4A**), the amount of cells with centrosomal JAK2 localization was less for both mutants as compared with WT JAK2 (**Fig 4F**). When we overexpressed a kinase-dead mutant JAK2 K882E (JAK2 K-E) (**Fig 4B**), it also decreased the amount of cells with centrosomal JAK2, confirming that the kinase activity participates in the centrosomal localization of JAK2. Because an arginine-426 within the SH2 domain is a common loss of function mutation used in SH2 domain studies [42–44], we mutated this arginine to alanine (R426A mutant) (**Fig 4D**). However, this point mutation did not affect JAK2 centrosomal localization as compared with the WT protein (**Fig 4F**). This observation confirms the previously published data that R426A mutant (or corresponding R466K in JAK1) does not alter the receptor-binding ability of JAK and/or downstream signaling [45–47] supporting the unconventional feature of the JAK SH2 domain (see Discussion). Finally, we created a double mutant of JAK2, lacking both the AA 381–544 and the kinase activity (ΔSH2, K882E mutant) (**Fig 4E**) and this mutant failed to localize to the centrosome (**Fig 4F**, last bar). Based on these data we concluded that AA 381–544 containing the SH2 domain and the kinase activity are necessary for centrosomal localization of JAK2.

### The same domains that target JAK2 to centrosome, regulate cell proliferation and cell cycle

**Fig 5:**
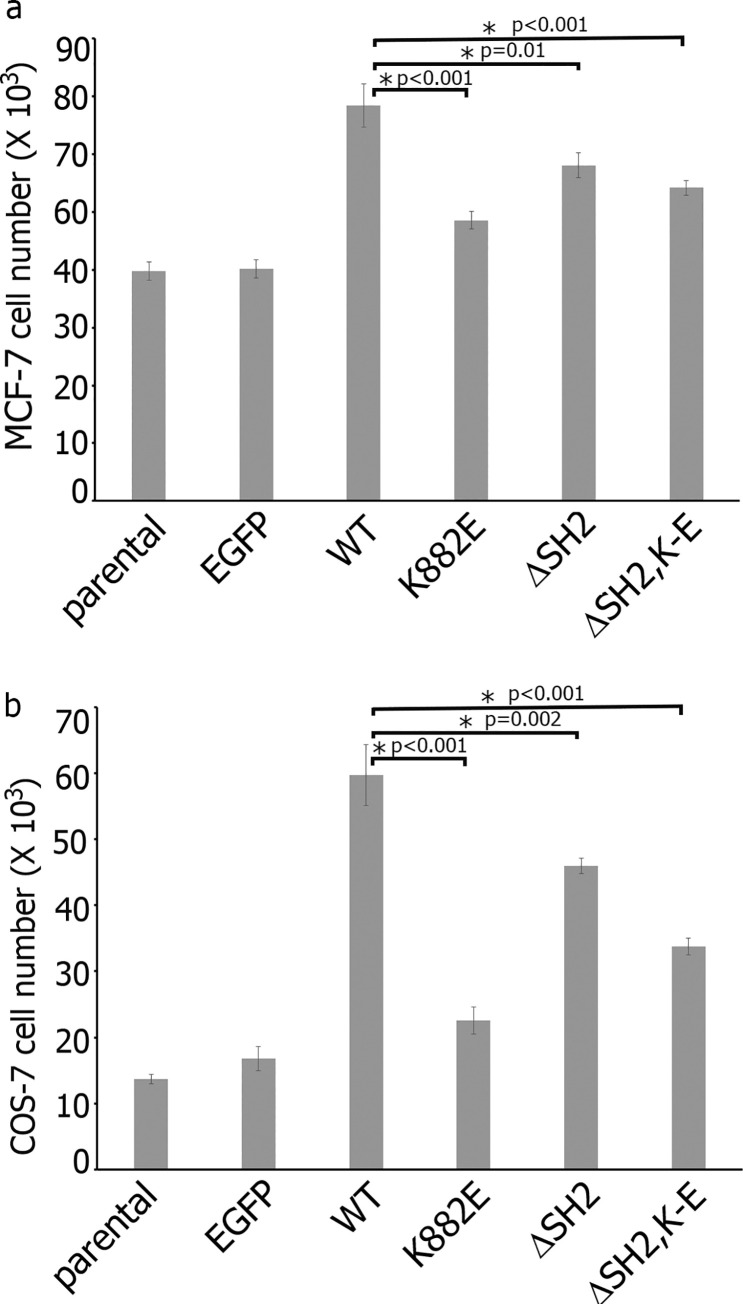
Proliferation of COS-7 and MCF-7 clones. (**a**) MCF-7 cells and COS-7 cells (**b**) (parental and stable clones overexpressing the indicated proteins) were plated at equal densities, deprived of serum for 48 hrs and then treated with 500 ng/ml PRL (MCF-7 cells) or 30 ng/ml EGF (COS-7 cells). The number of cells was counted in 4 days. Overexpression of WT JAK2 increases the cell proliferation while overexpression of JAK2 mutants does not. Bars represent mean ±SE. *, P< 0.05 compared with WT cells.

To determine the impact of domains that are responsible for centrosomal localization of JAK2 on JAK2-dependent cellular functions, we established COS-7 and MSF-7 cell lines that stably express EGFP either alone (as vector control) or fused with WT JAK2 or centrosomal-deficient JAK2 mutants: ΔSH2 (SH2 domain-deficient mutants), K882E (K-E, kinase dead mutants) and (ΔSH2, K-E) (SH2 domain-deficient and kinase dead double mutant) (**S1 and S2 Figs**). To determine whether JAK2 localization has a physiological impact on cells, we evaluated the effect of domains, responsible for centrosomal localization of JAK2, on cell proliferation because JAK2 has been implicated in proliferation of normal and malignant cells [48]. The JAK2 pathway plays a critical role in PRL-induced proliferation of normal mammary epithelial cells, as well as in human breast cancer cells [49–51], and we have previously shown that PRL induces proliferation of different breast cancer cells [52]. We deprived MCF-7 clones of serum, treated them with PRL and assessed the cell proliferation in 4 days. We have shown that the overexpression of JAK2 WT strongly enhanced PRL-dependent cell proliferation as compared to parental and EGFP expressing cells while JAK2 ΔSH2, K-E and (ΔSH2, K-E) clones proliferated to a lesser degree than JAK2 WT clone (**Fig 5A**). To confirm that this effect is not restricted to a single cell line, we assessed proliferation of COS-7 clones in response to EGF because EGF is a mitogen and also activates JAK2 pathways ([53, 54]; reviewed in [1, 55]). As shown in Fig 5B, overexpression of JAK2 WT strongly promoted EGFP-triggered cell proliferation while effect of overexpression of ΔSH2, K-E and (ΔSH2, K-E) mutants on cell proliferation was much weaker (**Fig 5B**).

**Fig 6:**
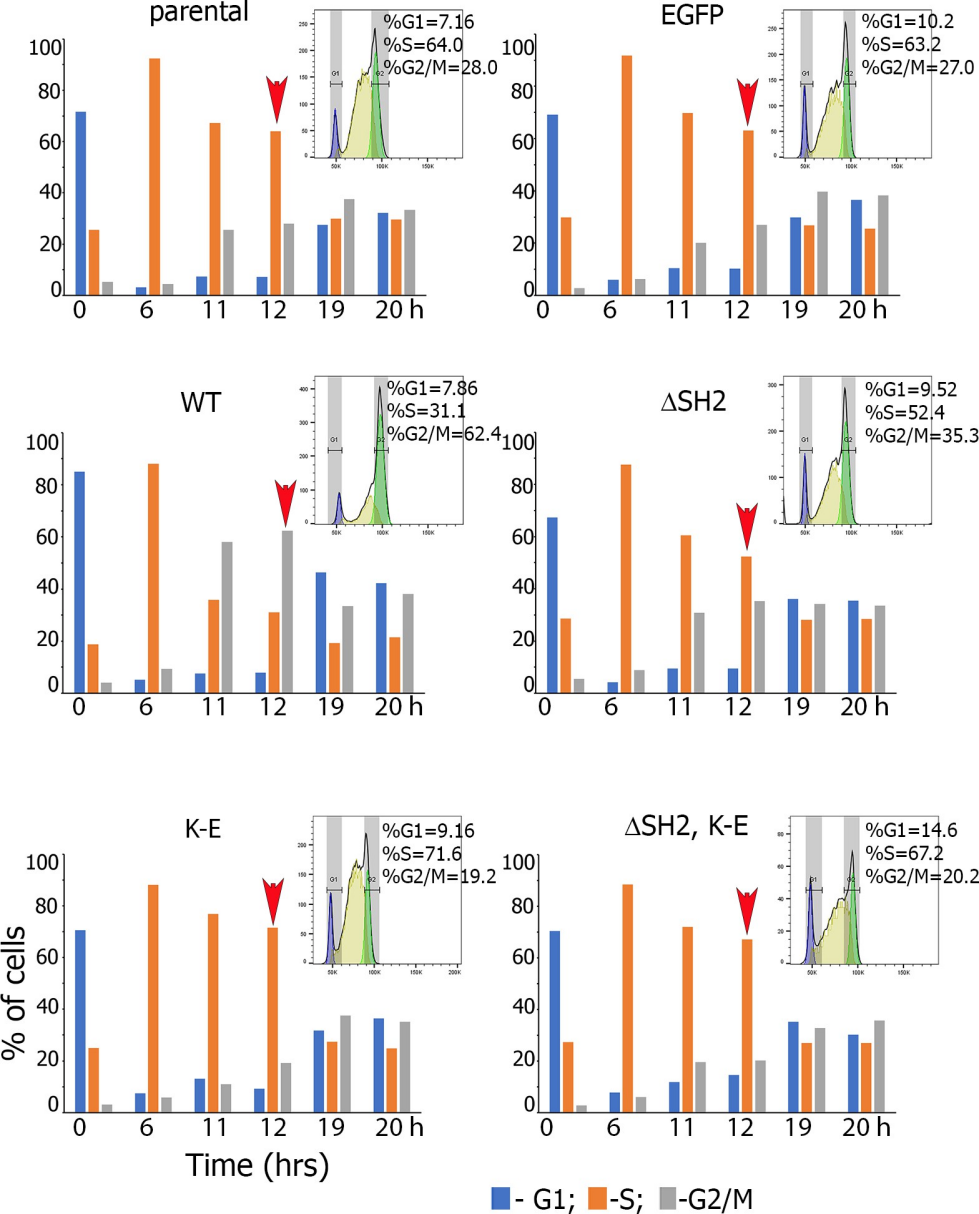
Cell cycle analysis of MCF-7 cell clones. Parental MCF-7 cells and the MCF-7 cell clones stably overexpressing indicated constructs were PRL-treated and synchronized at the G1/S boundary by single thymidine block. To determine the duration of S and G2/M phases, the cells were released, collected every 2 hrs and analyzed by FACS. In 12 hrs after the release only majority of JAK2 WT cells were in G2/M phase while parental, EGFP and mutant cells still stayed in S phase (arrowhead). The FACS analysis was repeated three times for each experimental condition with similar results. Figure demonstrates the representative experiment.

To explain our proliferation data and test whether the SH2 domain and the kinase activity alter the cell cycle, we synchronized PRL-treated MCF-7 clones in G1 by a single thymidine block. To determine the duration of S and G2/M phase, cells were collected every 2 hours and analyzed by FACS. Percentages of cells in different phases of the cell cycle were calculated using Flowjo by gating on G1, S, and G2/M cell populations. The maximum amount of all cells were in S phase in 6 hours after release (**Fig 6**). The majority of the parental cells (64%) and EGFP clone (63%) was still in S phase 12 hours after the release. In contrast, JAK2 WT clone progressed much faster through the cell cycle as compared to control cells, with 62% of cells already in G2/M in 12 hours. ΔSH2, K-E and (ΔSH2, K-E) mutants of progressed much slower through the cell cycle as compared to JAK2 WT clone with the majority of cells (52%, 72% and 67%, respectively) still in S phase at the 12 hour time point. The FACS data revealed that enhanced proliferation of JAK2 WT clone was accompanied by increased S −> G2 progression of these cells and that this effect depended on the SH2 domain and kinase activity of JAK2.

### SH2 domain of JAK2 affects STAT5 activation

**Fig 7:**
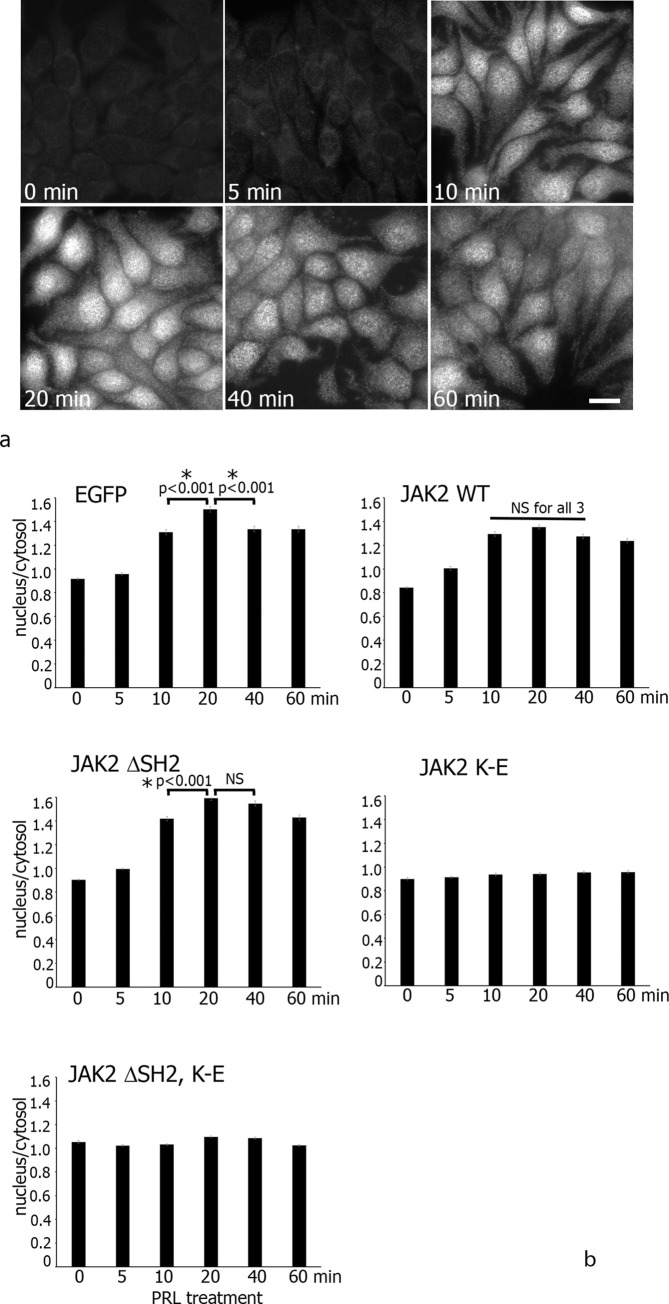
Kinase activity and the SH2 domain of JAK2 play a role in activation of STAT5 in response to prolactin. (**a**) MCF-7 cells were serum-deprived, treated with PRL for different time points and immunolabelled with anti-pSTAT5 AB. PRL causes maximal nuclear accumulation of pSTAT5 at 20 min. (**b**) The fluorescence intensity of the cytosol and nucleus in 50 cells per condition in 3 independent experiments was measured and the nuclear-to-cytosol fluorescence ratios (N/C) were calculated and plotted. Bars represent mean ±SE. *, P< 0.05. Scale bar 10 μm.

To determine the impact of domains targeting JAK2 to the centrosome on JAK2-dependent signaling, we assessed STAT5 activation. As prolactin activates JAK2 and JAK’s physiological substrate is STAT5, we used MCF-7 cells which have the PRL receptors. We treated stable MCF-7 clones expressing either EGFP or JAK2 WT and centrosomal-deficient JAK2 mutants (ΔSH2, K-E and (ΔSH2, K-E)) with PRL for different times, fixed the cells, and immunolabeled activated STAT5 with anti-phospho-STAT5 (pSTAT5) AB. As activated STAT5 translocates into the nucleus, we measured the fluorescence intensity of the cytosol and nucleus in 50 cells per condition (**Fig 7A**). **Fig 7B** shows the nuclear-to-cytosol fluorescence ratios (N/C) for three independent experiments. PRL stimulation of EGFP, WT JAK2 and ΔSH2 clones resulted in a dramatic nuclear accumulation of pSTAT5 in the majority of cells (>90%). In EGFP clone the maximal nuclear accumulation of pSTAT5 was detectable at 20 minutes of PRL treatment, while the overexpression of WT JAK2 shows no statistical difference between 10, 20 and 40 minute time points, which suggested that the overexpression of WT JAK2 caused pSTAT nuclear translocation faster (already at 10 minutes) and persisted longer (by 40 minutes of treatment) as compared to EGFP clone. Interestingly, ΔSH2 JAK2 activated STAT5 to the maximal extent slower than JAK2 WT (20 minutes vs. 10 minutes), although this prominent nuclear accumulation persisted as long as in JAK2 WT clone (40 minutes). These data suggest that the SH2 domain of JAK2 plays a role in STAT5 activation. As expected, both K-E and (ΔSH2, K-E) clones failed to activate STAT5 because they overexpress the kinase-dead JAK2 K-E mutant and the kinase activity of JAK2 is required for STAT5 activation. To test whether point mutation R426A in the SH2 domain of JAK2 affects STAT5 activation, we transiently transfected MCF-7 cells with either JAK2 R426A or with JAK2 WT, treated with prolactin for 20 min and assessed the nuclear translocation of pSTAT5 by anti-pSTAT5 immunolabeling. R426A and WT JAK2 expressing cells demonstrated a similar amount of cells with nuclear pSTAT5 (73.0% and 73.3% respectively) suggesting that R426A mutation does not affect STAT5 activation as compared with the JAK2 WT protein (S3 Fig). Similarly, the R426A mutation of JAK2 and the corresponding R466K mutation in JAK1 had no effect on STATs activation [45, 46].

### JAK2 prevents centrosome amplification in SH2-domain-dependent manner

**Fig 8:**
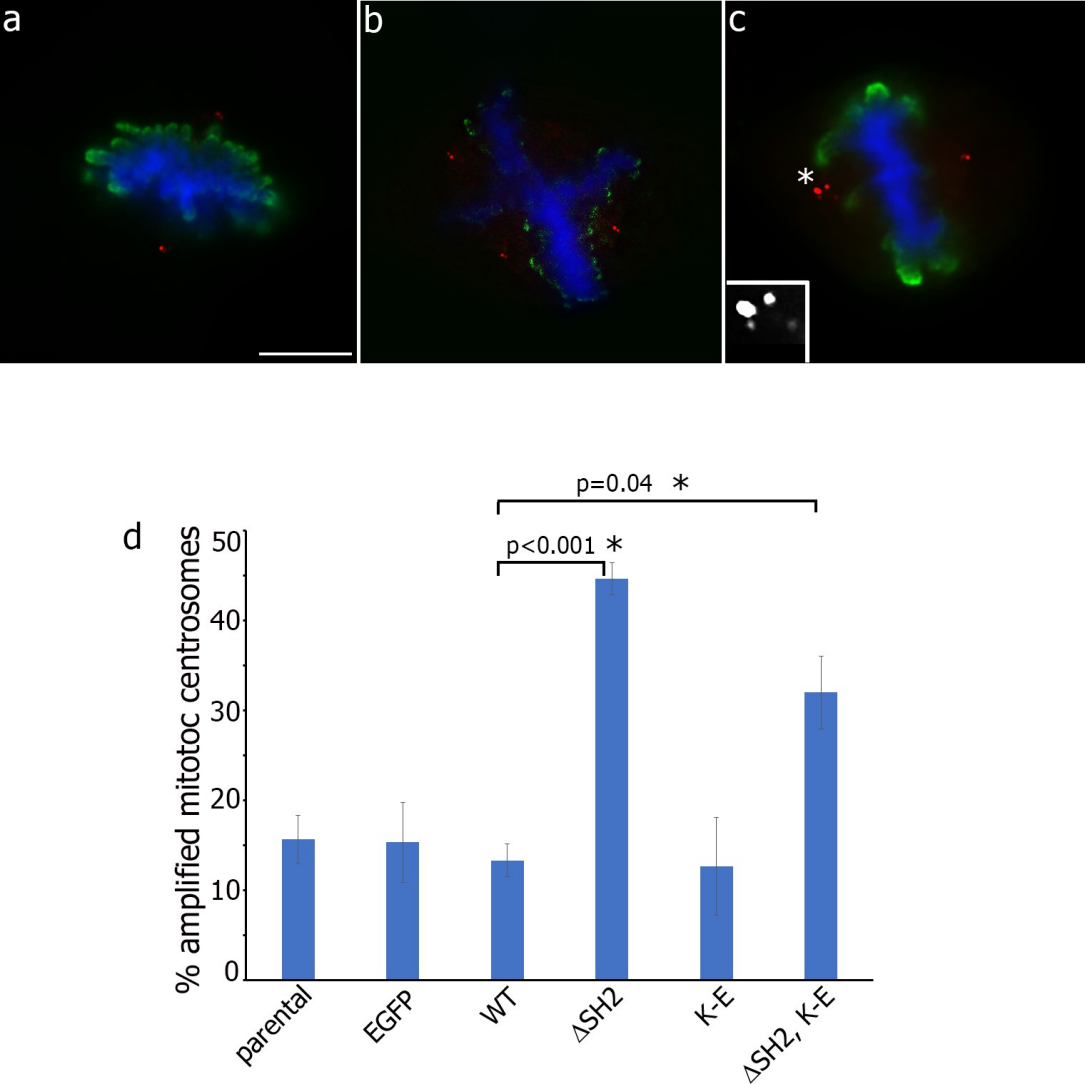
JAK2 regulates centrosome amplification and the SH2 domain of JAK2 is required for that. Parental COS-7 cells and stable COS-7 clones were fixed, stained with DAPI (blue) and immunolabeled with anti-pHistone H3 (green) for assessment of mitotic cells only. Centrosomes were immunolabeled with anti-centrin 1 (red). The cells were scored for (**a**) normal mitosis with ≤ 2 centriolar pairs, mitosis with amplified centrosomes (>2 centriolar pairs), including multipolar mitosis (**b**) and mitosis with clustered centrosomes (**c**). The inset in (c) shows enlarged centrosomes. (**d**) The graph shows the percentage of cells with amplified centrosomes (50 mitotic cells per experiment, 3 independent experiments for each cell clone and parental cells). Bars represent mean ±SE. *, P< 0.05 compared with WT cells. Scale bar 8 μm.

We have previously demonstrated that JAK2-deficient γ2A cells exhibit more mitotic defects as compared to the 2C4 cells (syngeneic parental cells that express wild type JAK2) [18]. The most significant mitotic defect in γ2A cells was the increased incidence of lagging chromosomes. This defect usually results from the aberrant attachment of one kinetochore to both spindle poles due to spindle defects caused by centrosome amplification [18, 20, 56]. Therefore, we examined whether domains targeting JAK2 to the centrosome can affect centrosome amplification. Supernumerary centrosomes lead to formation of multipolar spindles (**Fig 8B**). However, cells can use a mechanism to divide with excess centrosomes: they cluster their centrosomes together into 2 foci to generate a pseudo-bipolar spindle structure [57]. We assessed the amount of centrosomes in mitotic cells stained with DAPI and the mitotic marker phospho-histone H3. We accounted cells with ≤ 2 centriolar pairs (“normal”, **Fig 8A**), and >2 (clustered or not) centriolar pairs (“amplified”, **Fig 8B and 8C**). Parental COS-7 cells, EGFP and WT clones demonstrated a similar amount of cells with multiple centrosomes (15.7%, 15.3% and 13.3% respectively) (**Fig 8D**). Interestingly, the kinase activity of JAK2 is not involved in JAK2-depending regulation of centrosome amplification, because JAK2 K-E mutant exhibited the same percentile of cells with supernumerary centrosomes as WT JAK2 (12.7%), while both ΔSH2 JAK2 and double mutant (ΔSH2, K-E) sufficiently increased the amount of cells with amplified centrosomes (44.7% and 32% respectively) compared to the WT cells (13%). These data suggest that the SH2 domain of JAK2 plays a role in regulation of centrosome amplification. To test whether point mutation R426A in the SH2 domain of JAK2 affects centrosome amplification, we transiently transfected COS-7 cells with either JAK2 R426A or with JAK2 WT and assessed the amount of centrosomes in mitotic cells as described above. As expected, R426A did not affect centrosome amplification as compared with the JAK2 WT protein (**S4 Fig**).

## Discussion

We have previously shown that endogenous tyrosine kinase JAK2 localizes to the mother centriole in all phases of the cell cycle [18]. Here we have extended these data and demonstrated that centrosomal localization of JAK2 depends on AA (381–544), containing the SH2 domain, and JAK2 kinase activity. We have shown that kinase-dead mutant K882E (JAK2 K-E) and a mutant lacking the SH2 domain (JAK2 ΔSH2) have impaired centrosomal localization, while the double-mutant JAK2 (ΔSH2, K882E or ΔSH2, K-E) is unable to localize to centrosomes. These domains of JAK2 play a role in cell growth, as the clones overexpressing ΔSH2, K-E and (ΔSH2, K-E) mutants proliferate in response to prolactin slower than the cells overexpressing JAK2 WT. The cell cycle analysis reveals that all three mutant clones (ΔSH2; K-E and (ΔSH2, K-E)) progress much slower through the cell cycle compared to JAK2 WT clone, demonstrating S-phase delay while the enhanced proliferation of JAK2 WT clone is accompanied by accelerated S −> G2 progression. As the STAT5 is a physiological substrate of PRL-activated JAK2, we have shown that the SH2 domain of JAK2 plays a role in STAT5 activation. The SH2 domain of JAK2 also regulates centrosome biogenesis. Thus, ΔSH2 and (ΔSH2, K-E) mutants strongly increased centrosome amplification. In contrast, the kinase activity of JAK2 does not play a role in this process.

JAK proteins contain four structural domains: the N-terminal FERM (Band 4.1, Ezrin, Radixin, Moesin-homology) domain, the SH2 domain, the pseudokinase domain, and the C-terminal catalytic (kinase) domain (**Fig 1**). The FERM and SH2 domains are necessary for the JAKs interaction with the cytoplasmic tails of the receptors, which contain “box1” and “box2” motifs required for JAK engagement (reviewed in [58]). Interestingly, the SH2 domain in JAK kinases does not maintain the phosphotyrosine (pTyr)-binding function of classical SH2 domains [47, 59]. This unconventional feature of the JAK SH2 domain was first demonstrated in early studies, which found no phenotype with mutation of a key conserved arginine in this domain [45–47]. Consistent with these studies, solved crystal structure of the JAK2 FERM-SH2 fragment revealed that the canonical phosphotyrosine pocket is blocked in the SH2 domain, therefore this domain cannot recognize phosphorylated tyrosines [60]. Interestingly, the SH2 domain-dependent but pTyr-independent interaction has been described between many proteins including Vav1 and the Mer receptor tyrosine kinase [61], SOCS-1 and MTOC-associated 20S proteasome [62], SAP protein and lymphocyte coreceptor SAP [63] and between integrin β and tyrosine kinases Syk and ZAP-70 [64]. What could be the functions of this non-classical SH2 domain in JAK2? Surprisingly, not much is known about the function of the SH2 domain in JAK kinases (reviewed in [58, 65]). This domain has been shown to be necessary for receptor interaction and JAK2 activation, as the oncogenic JAK2 V617F mutation within the pseudokinase domain, found in myeloproliferative neoplasms, requires an intact SH2 domain [47, 59]. Additionally, the JAK SH2 domain mediates not only receptor binding, but also receptors’ Golgi processing and cell surface expression [47, 66]. Here we propose a novel function of the JAK2 SH2 domain that is centrosomal localization of JAK2 and regulation of centrosome amplification.

The centrosome is not only the main MT-nucleating organelle in animal cells that regulates the organization of the interphase cytoskeleton and mitotic spindle. The centrosome is a multifunctional complex that contains about 200 core centrosome proteins and nearly 600 associated proteins based on a combination of two current databases (Centrosome:db [67] and MiCroKit3.0 [68] and proteomic studies ([69–72]; reviewed in [22–24, 73, 74]). Moreover, novel centrosomal proteins are still being discovered. Many proteins found at the centrosomes are cyclins, various kinases, phosphatases, signaling substrates, and cell cycle regulators. Thus, the centrosome serves as a scaffold for anchoring hundreds of regulatory proteins, some only transiently and others throughout the cell cycle [22, 25, 26, 28–30]. A variety of JAK2 regulators and substrates, including SOCS-1, Fyn kinase, PI3K, protein phosphatases PTP-BL and SHP-1, and BRCA1, have been detected at the centrosome [62, 75–82]. STATs 1, 3, 5A and 5B, all of which are JAK2 substrates, have been also found on centrosomes [27, 83].

It is known that both the intensity and duration of the STAT response are tightly regulated and inappropriate STAT activation leads to oncogenesis [84]. It has been suggested that the specificity and diversity in STAT signaling pathways is dictated by multiple factors and one of them is STATs localization on centrosomes. Thus, c*entrosomal* P4.1-associated protein (CPAP) acts as a *STAT5* coactivator to enhance PRL-triggered transcription [85]. Phosphorylation, nuclear localization and transcriptional activity of STAT3 is regulated by Aurora A kinase which is present on duplicated centrosomes from late S phase until early G1 phase [86–88]. We have shown here that PRL-dependent STAT5 activation was weaker in ΔSH2 JAK2 clone with impaired centrosomal localization than in the JAK2 WT clone. We hypothesize that localization of both WT JAK2 and STAT5 on centrosomes may bring them together to enhance STAT5 activation.

It has been shown that STATs reciprocally regulate centrosomal biogenesis. Thus, STAT5 induces centrosomal duplication throughout inducing expression of Aurora A [89]. STAT3 is also required for centrosome duplication [90] and interacts with and inhibits the MT depolymerase Stathmin [91]. STAT3 is also involved in the regulation of supernumerary centrosome clustering via Stathmin/PLK1 [92]. In contrast, recent data have shown that STAT3 inhibits centrosomal amplification via JNK1 pathway [93], demonstrating that mechanisms responsible for the effect of STATs on centrosomal amplification remain elusive. These issues have not been investigated here, and will be addressed in detail in our planned future studies. Instead, in the current study we have shown an important role of SH2 domain of JAK2 in centrosome amplification. In proliferating cells, centrioles duplicate every cell cycle by forming one new procentriole adjacent to each existing parent centriole. Recent years have seen an explosion of interest in understanding how cells maintain centriole copy number through successive cell cycles [94]. Like centrosome loss, centrosome amplification also suppresses the proliferation of cells in culture by different mechanisms (reviewed in [95]). We have shown a correlation between the centrosomal localization of JAK2, cell proliferation and centrosome amplification. Thus, the SH2-domain-deficient clones ΔSH2 and (ΔSH2, K-E) demonstrate the low proliferation and the high amount of amplified centrosomes.

Interestingly, the JAK2 K882E mutant did not affect centrosome amplification. Accumulating evidences suggest that some JAK2 functions can be kinase-independent. While JAK2 kinase activity plays a major role in JAK2 downstream signaling events, JAK2 can also act independently of its kinase activity as a molecular scaffold to facilitate the interaction between different proteins. Thus, the kinase-inactive JAK2 mediates biologically relevant activation downstream signaling in mice expressing an activation-loop mutant allele (YY1107/1008FF) (the tandem tyrosines 1007/1008 in the activation loop of the kinase domain are required for the kinase activity of JAK2). These data demonstrate that JAK2 can act *in vivo* as a scaffold protein independent of its kinase activity [96]. JAK2 also serves as the scaffold to couple a G protein-dependent angiotensin II pathway and STATs pathway (reviewed in [97]). Moreover, kinase-dead or inhibited JAK2 can serve as the scaffold for transactivation by other JAK kinases [98]. These non-catalytic functions were described for the growing list of different protein kinases (reviewed in [99]). We can speculate that the SH2 domain of kinase-dead JAK2 binds to other proteins at the centrosomes to regulate the centrosomal amplification. It would explain why SH2 domain but not kinase activity regulates centrosome amplification. Which JAK2 partners participate in centrosomal biogenesis remains to be explored.

In summary, we report that localization of JAK2 on centrosome depends on the SH2 domain and the JAK2 kinase activity. The same domains of JAK2 play an important role in PRL-dependent cell proliferation, cell cycle control, activation of STAT5 and centrosomes amplification. Thus, data presented here bring significant new insight into the role of SH2 domain and kinase activity of JAK2 in centrosome localization of JAK2 and in the regulation of cell growth and centrosome biogenesis.

## Supporting information

S1 Fig Characterization of COS-7 and MCF-7 cell lines stably expressing EGFP alone, or EGFP-tagged JAK2 WT and JAK2 mutants. Parental COS-7 (a) and MCF-7 (b) cells, the cell clones stably expressing EGFP, EGFP-JAK2 WT, and indicated mutants of JAK2 were lysed, and proteins were resolved by SDS-PAGE. Overexpressed proteins were visualized by immunoblotting with anti-GFP and anti-JAK2 AB. Asterisk (*) indicates longer exposure with αJAK2 revealing endogenous JAK2. The expression levels of γ-tubulin were used as an internal control. The same membranes were incubated with anti-JAK2, anti-GFP and anti-γ-tubulin AB. The cropped blots are shown.
(TIF)

S2 Fig Raw images of blots of COS-7 and MCF-7 cell lines stably expressing EGFP alone, or EGFP-tagged JAK2 WT and JAK2 mutants. These full length raw images were used to generate S1 Fig. The lines not included in S1 Fig are marked “X”. The original films were scanned using an Epson Perfection V800 Photo scanner and saved as tiff files.
(TIF)

S3 Fig Point mutation R426A in the SH2 domain of JAK2 does not affects STAT5 activation in response to prolactin. MCF-7 cells were transiently transfected with cDNA encoding either EGFP-JAK2 WT or EGFP-JAK2 R426A. The cells were serum-deprived, treated with prolactin (500 ng/ml) for 20 min, fixed and immunolabelled as in Fig 7. A—pSTAT5 (red in images) accumulated in the nucleus of both EGFP-JAK2 WT and EGFP-JAK2 R426A cells (green in images). B—The graph shows the percentage of cells with nuclear pSTAT5 (50 EGFP-positive cells per experiment, 3 independent experiments). Number of pSTAT5-positive nuclei were assessed in EGFP-positive cells only. Bars represent mean ±SE.
(TIF)

S4 Fig Point mutation R426A in the SH2 domain of JAK2 does not affects centrosome amplification. COS-7 cells were transiently transfected with cDNA encoding either EGFP-JAK2 WT or EGFP-JAK2 R426A. The cells were fixed and immunolabelled as in Fig 8. Mitotic centrosomes were assessed in EGFP-positive cells only. The graph shows the percentage of cells with amplified centrosomes (50 mitotic cells per experiment, 3 independent experiments). Bars represent mean ±SE.
(TIF)
