# Supplementary figures and images for: The SH2 domain and kinase activity of JAK2 target JAK2 to centrosome and regulate cell growth and centrosome amplification
Source: PLoS One. 2022 Jan 28;17(1):e0261098. doi: 10.1371/journal.pone.0261098 (PMC8797172; doi:10.1371/journal.pone.0261098)

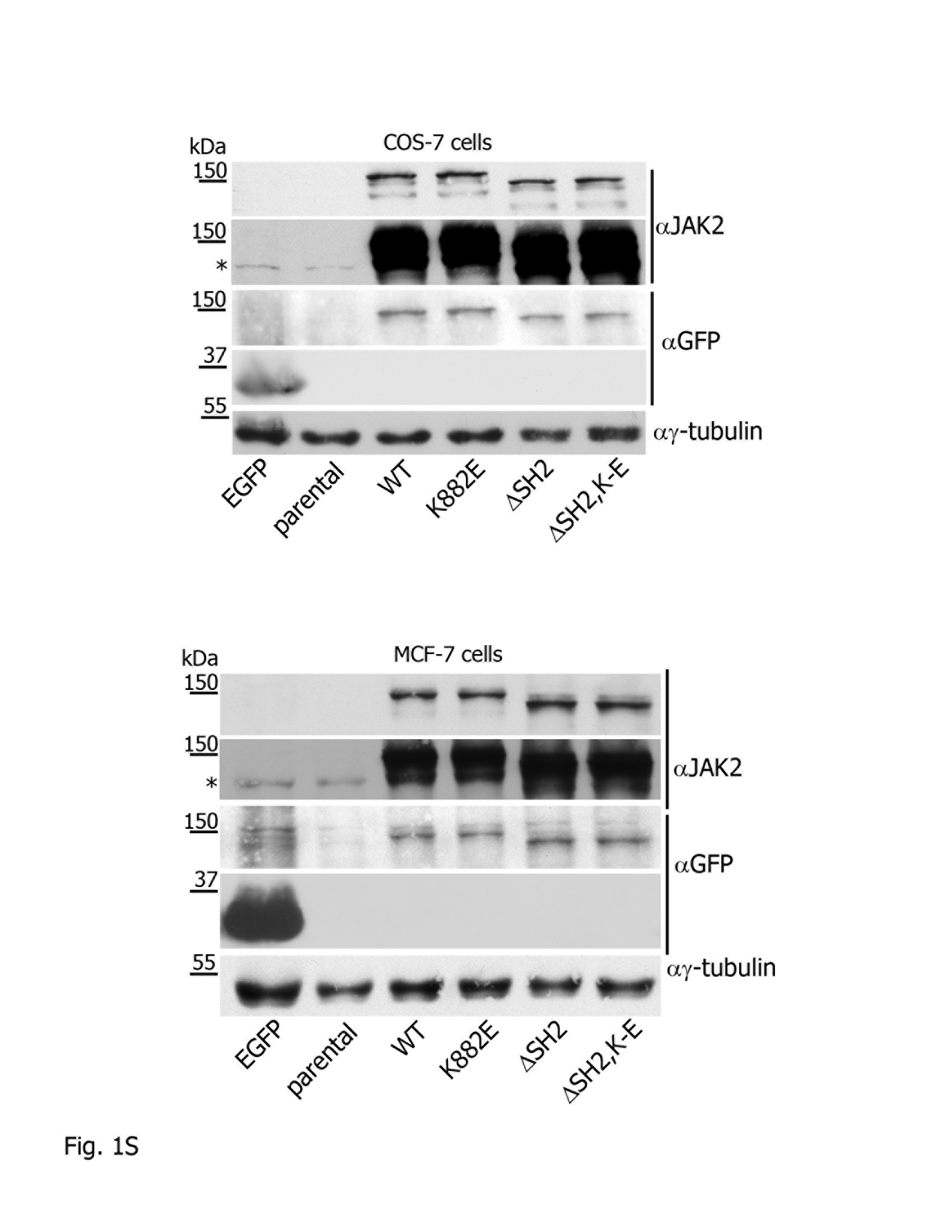

Supplement: S1 Fig — Parental COS-7 (a) and MCF-7 (b) cells, the cell clones stably expressing EGFP, EGFP-JAK2 WT, and indicated mutants of JAK2 were lysed, and proteins were resolved by SDS-PAGE. Overexpressed proteins were visualized by immunoblotting with anti-GFP and anti-JAK2 AB. Asterisk (*) indicates longer exposure with αJAK2 revealing endogenous JAK2. The expression levels of γ-tubulin were used as an internal control. The same membranes were incubated with anti-JAK2, anti-GFP and anti-γ-tubulin AB. The cropped blots are shown. (TIF) [file pone.0261098.s001.tif]

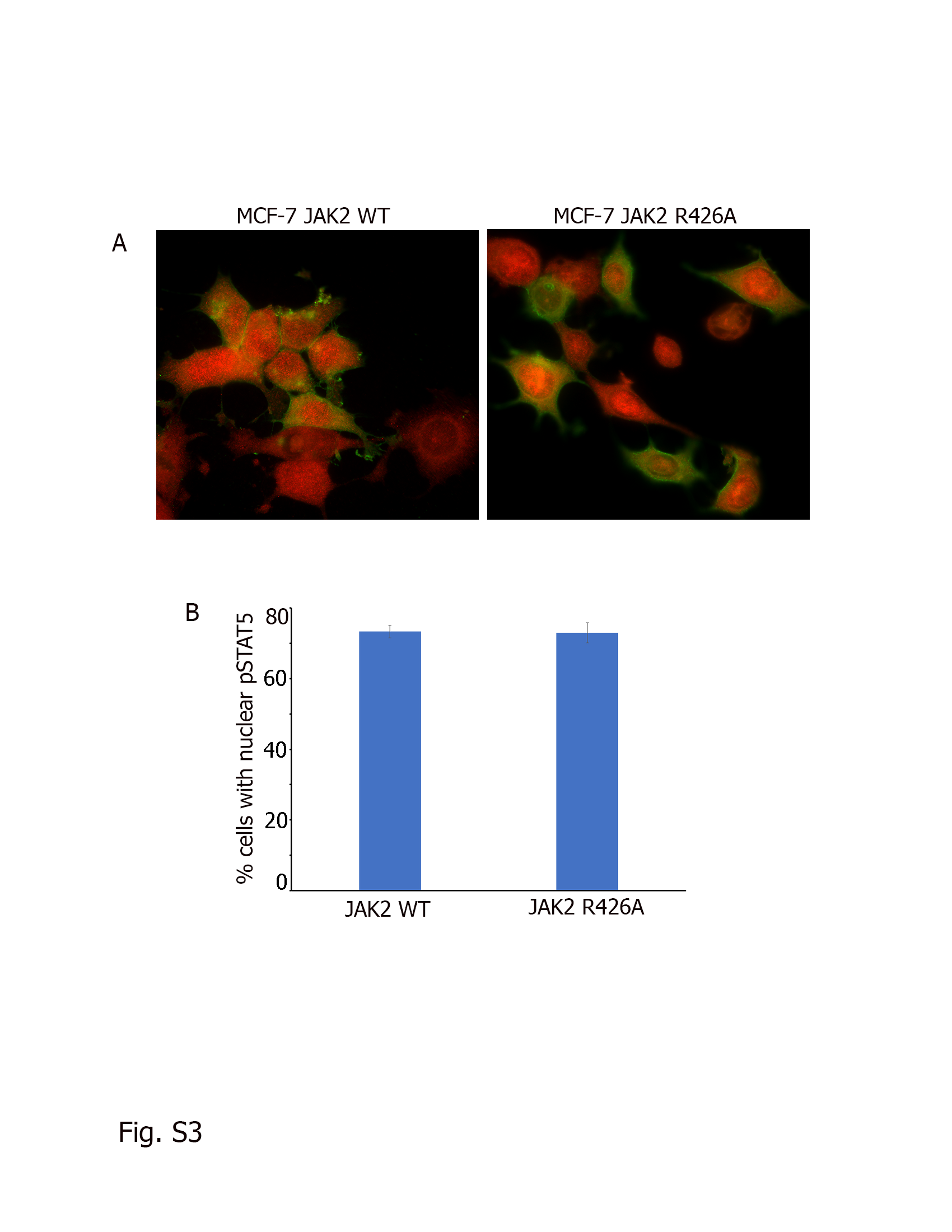

Supplement: S3 Fig — MCF-7 cells were transiently transfected with cDNA encoding either EGFP-JAK2 WT or EGFP-JAK2 R426A. The cells were serum-deprived, treated with prolactin (500 ng/ml) for 20 min, fixed and immunolabelled as in Fig 7. A—pSTAT5 (red in images) accumulated in the nucleus of both EGFP-JAK2 WT and EGFP-JAK2 R426A cells (green in images). B—The graph shows the percentage of cells with nuclear pSTAT5 (50 EGFP-positive cells per experiment, 3 independent experiments). Number of pSTAT5-positive nuclei were assessed in EGFP-positive cells only. Bars represent mean ±SE. (TIF) [file pone.0261098.s003.tif]

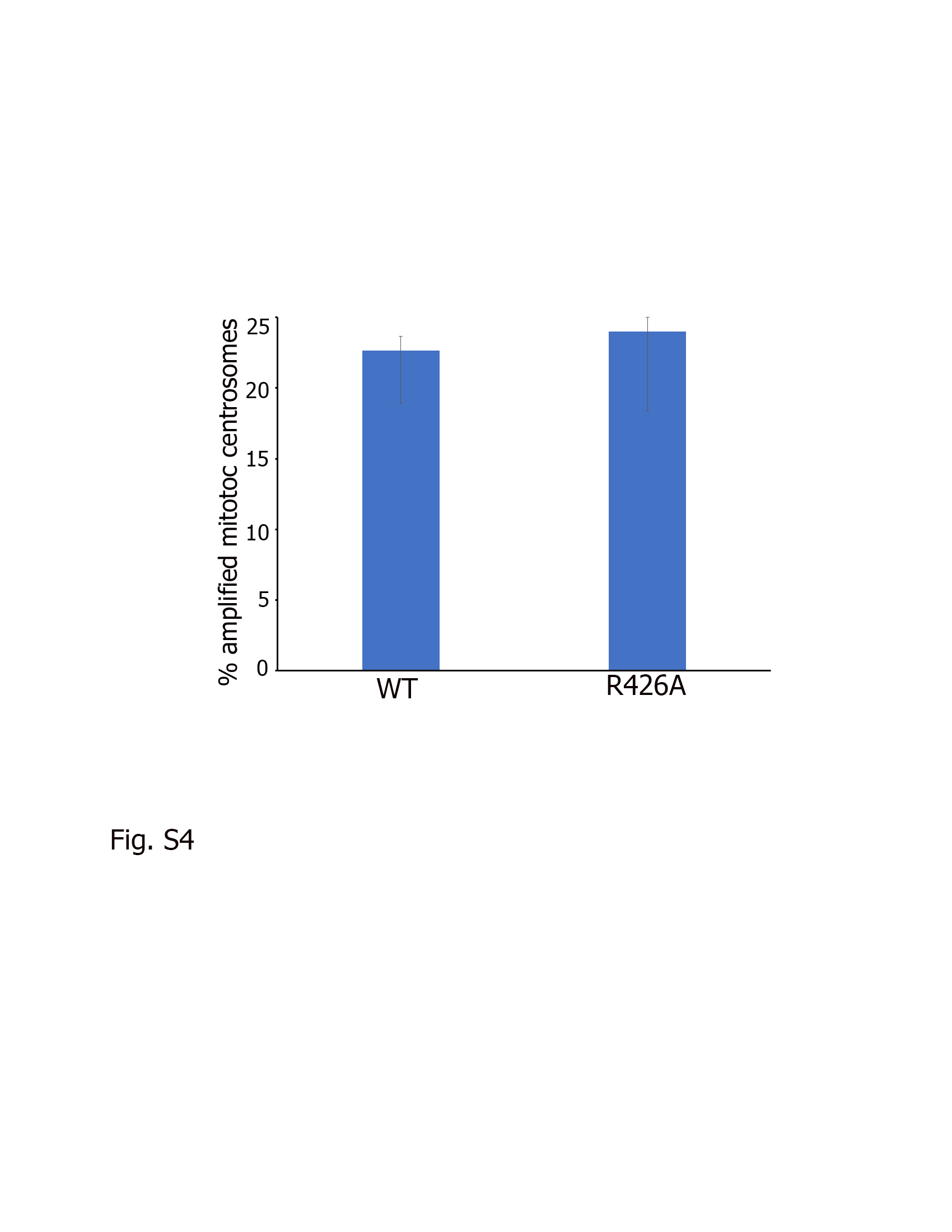

Supplement: S4 Fig — COS-7 cells were transiently transfected with cDNA encoding either EGFP-JAK2 WT or EGFP-JAK2 R426A. The cells were fixed and immunolabelled as in Fig 8. Mitotic centrosomes were assessed in EGFP-positive cells only. The graph shows the percentage of cells with amplified centrosomes (50 mitotic cells per experiment, 3 independent experiments). Bars represent mean ±SE. (TIF) [file pone.0261098.s004.tif]
